# Liquid Phase Catalytic Transfer Hydrogenation of Crotonaldehyde over ReO_x_-Supported Catalysts Using Formic Acid as In Situ Hydrogen Donor

**DOI:** 10.3390/molecules30214307

**Published:** 2025-11-05

**Authors:** Carlos Esteban Aristizábal-Alzate, Verónica Naharro-Ovejero, Manuel Romero-Sáez, Ana Belén Dongil

**Affiliations:** 1 Grupo Química Básica, Aplicada y Ambiente-Alquimia, Facultad de Ciencias Exactas y Aplicadas, Instituto Tecnológico Metropolitano (ITM), Medellín 050034, Colombia; carlosaristizabal4808@correo.itm.edu.co; 2 Grupo de Química Verde y Catálisis, Instituto de Catálisis y Petroleoquímica, Consejo Superior de Investigaciones Científicas (CSIC), Cantoblanco, 28049 Madrid, Spain; v.naharro@csic.es

**Keywords:** selective hydrogenation, heterogeneous catalysis, rhenium, unsaturated aldehyde, unsaturated alcohol

## Abstract

The selective hydrogenation of the C=O bond over the C=C bond in α,β-unsaturated aldehydes remains a well-known challenge. This work investigates the liquid-phase catalytic transfer hydrogenation of crotonaldehyde to crotyl alcohol over ReOx-based catalysts, using formic acid (FA) as an in situ hydrogen donor. A series of 10 wt% Re catalysts supported on G200, g-C_3_N_4_, TiO_2_, and ZrO_2_ were synthesized and tested in a batch reactor at 20 bar and temperatures of 140–180 °C. Catalysts were characterized by XRD, BET, NH_3_-TPD, and XPS to correlate their physicochemical properties with catalytic behavior. Among the studied materials, ReO_x_/ZrO_2_ and ReO_x_/g-C_3_N_4_ exhibited the highest crotyl alcohol selectivity above 57% for all reaction temperatures, evaluated at crotonaldehyde conversion of 25%. The nature of the support strongly influenced the dispersion and oxidation state of Re species, as well as the surface acidity, which governed the activation of both the carbonyl group and the FA decomposition. Compared with molecular hydrogen, FA improved both conversion and selectivity due to its superior hydrogen-donating ability in the aqueous phase. These findings demonstrate that tailoring the acid–base characteristics of ReO_x_ catalysts and employing biomass-derived hydrogen donors represent an effective strategy for selective hydrogenation of α,β-unsaturated aldehydes.

## 1. Introduction

The hydrogenation of unsaturated aldehydes, such as crotonaldehyde, into unsaturated alcohols using heterogeneous catalysts has attracted great interest because these compounds serve as key intermediates in the industrial synthesis of fragrances, flavoring agents, and pharmaceutical products [1,2]. Among these chemoselective hydrogenation processes, the reduction in α,β-unsaturated aldehydes to allyl alcohols stands out as particularly challenging. The formation of saturated aldehyde is both thermodynamically and kinetically favored over the hydrogenation to the unsaturated alcohol due to the higher susceptibility of the C=C bond to hydrogenation (578.8 kJ mol^−1^) compared to the C=O bond (705.8 kJ mol^−1^) [3,4]. Therefore, heterogeneous catalysts that are selective towards the C=O bond in crotonaldehyde must be employed to improve the selectivity of allyl alcohol. In the present research, crotonaldehyde is selected as the substrate molecule to study its liquid phase hydrogenation over oxidized rhenium species (ReO_x_) supported catalysts. Rhenium has been selected because it exhibits remarkable versatility, as it can assume various roles depending on its oxidation state and the nature of the support, such as an active phase, promoter, or electronic modifier [5,6,7]. The predominant effects attributed to ReO_x_ as promoters in catalysts are based on their acidity and affinity for oxygen, which facilitate the operation of bifunctional reaction mechanisms and additional interactions with functional groups of adsorbed substrate molecules [7]. Therefore, it is expected that Re oxides are well-suited for enhancing selectivity towards crotyl alcohol.

Previous studies have evaluated the liquid-phase reaction of crotonaldehyde using noble metals more expensive than rhenium, such as Rh, Pt, or Ir, achieving selectivity to crotyl alcohol of 80–90% [8,9]. Tamura et al. [6]. evaluated Ir–ReO_x_ catalysts supported on SiO_2_, TiO_2_, and ZrO_2_ for crotonaldehyde hydrogenation under mild conditions (3.0 mmol of crotonaldehyde, 3.0 g of H_2_O, 50 mg catalyst with Re/Ir = 1, 30 °C, 1 h, 8 bar H_2_). The catalytic results revealed that the support influenced selectivity and conversion. Thus, with SiO_2,_ the reaction achieved 43.3% conversion and 95% selectivity toward crotyl alcohol, while TiO_2_ provided the same conversion (43.2%) but a lower selectivity (85.3%), and ZrO_2_ yielded 38.7% conversion and an intermediate selectivity (89.6%). These findings show that the support nature modulates the Re–Ir interface and, consequently, the hydrogenation pathway. On the other hand, the study mentions a pretreatment of the catalysts within the batch reactor at 200 °C and H_2_ at 80 bar per hour, and they utilized Ir, which, as previously mentioned, is more expensive than Re. In addition, carbon-supported noble-metal catalysts (Pt/C, Ru/C, Rh/C, Pd/C) and M-ReO_x_/SiO_2_ (where M = Pt, Ru, Rh, Pd; Re/M = 0.5) were applied to the reaction at a high H_2_ pressure of 8 MPa, resulting in low selectivity to crotyl alcohol of less than 10% and 35%, respectively [5].

The production of molecular hydrogen is highly energy-consuming, and it results in significant carbon dioxide emissions into the atmosphere [10,11]. This issue has prompted the use of alternative hydrogen sources. Moreover, hydrogen donors can increase the selectivity towards unsaturated alcohol, and in situ liberated hydrogen is more reactive than ex situ H_2_ sources [12]. Therefore, it is highly desirable to develop new strategies that can reduce the reliance on externally generated hydrogen for these processes [13,14,15]. For instance, Ye et al. [10]. reported that the hydrogenation of furfural to furfuryl alcohol using H_2_ achieves a selectivity of 4.7%, whereas using an in situ hydrogen donor, such as isopropanol, resulted in a selectivity of 89.6%. Nevertheless, the use of alcohol leads to an undesirable side effect: the formation of acetals and hemiacetals [16]. In gas-phase furfural hydrogenation, both furfural conversion and furfuryl alcohol selectivity are significantly higher when formic acid (FA) is used as the hydrogen donor instead of 2-propanol [12,17,18,19]. On the other hand, Du et al. [20]. reported 100% furfural conversion and 98.1% furfuryl alcohol selectivity in the liquid phase using FA as a hydrogen donor.

Despite the limited research on the hydrogenation of crotonaldehyde using FA as a hydrogen donor in aqueous phase, it has been demonstrated that the gas-phase process and the use of a rhenium-supported catalyst achieve medium-high conversion (35–95%) and selectivity toward unsaturated alcohol between 60 and 80% at 140 and 180 °C [21]. We selected FA as an in situ hydrogen donor since it is a biomass-derived product. Additionally, the reduction of the carbonyl group by using this organic acid facilitates the operability, reduces the cost of the process, and addresses safety concerns about handling high-pressure hydrogen gas [12,20,22]. For these reasons, this study analyzes the impact of FA as a hydrogen donor on crotonaldehyde hydrogenation in the liquid phase.

## 2. Results and Discussion

### 2.1. Catalysts Characterization

Table 1 displays the BET area for supports and catalysts, and the crystal size for ReO_x_ synthesized catalysts.

Two distinct trends were observed in the BET surface area after Re impregnation and thermal treatment. For metal oxide supports (TiO_2_ and ZrO_2_), the surface area remained nearly unchanged, indicating that ReO_x_ species were well dispersed without significant pore blockage or structural collapse. This behavior is consistent with the results reported by Bassi et al. [23], who observed that the high thermal stability of these supports preserves their textural properties after Re loading. Conversely, carbon-based supports (G200 and g-C_3_N_4_) exhibited a marked loss in surface area after impregnation and thermal treatment, more pronounced for g-C_3_N_4_, probably due to partial pore blockage [24,25].

**Figure 1:**
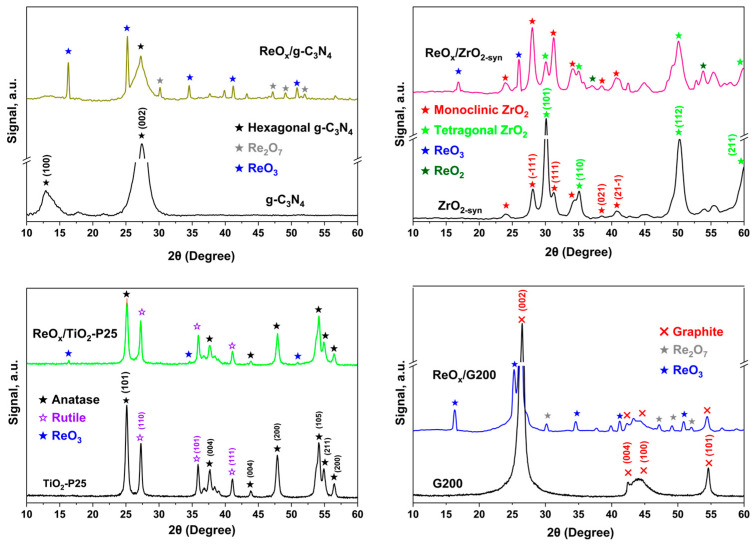
XRD patterns for ReO_x_-supported catalysts and supports.

Figure 1 shows the XRD for each ReO_x_ catalyst and its respective support. The pattern of g-C_3_N_4_ displayed a diffraction peak at 27.3°, ascribed to the hexagonal phase of g-C_3_N_4_, associated with the stacking of the conjugated aromatic system, identified as the (002) peak for graphitic materials and aligning with the interlayer d-spacing (0.336 nm) of g-C_3_N_4_, confirming the formation of g-C_3_N_4_. A weaker diffraction peak at 13.04° is indexed as (100) and corresponds to the tri-s-triazine units in the same plane [26,27]. The pattern obtained for ZrO_2_ includes diffractions at 24.07°, 28.06° (111), 31.2° (111), 34.1°, 38.4° (021), and 40.7° (211) for the monoclinic phase [28,29,30]. The ZrO_2_ tetragonal phase is also identified with peaks at 30.1° (101), 35.0° (110), 50.3° (112), and 59.8° (211). For TiO_2_-P25, XRD peaks at 25.1°, 37.7°, 43.8°, 47.8°, 54.1°, 54.9°, and 56.4° match with (101), (004), (004), (200), (105), (211), and (200) diffraction planes of anatase, respectively [31,32]. On the other hand, those located at 27.2°, 35.8°, and 41.06° are assigned to (110), (101), and (111) rutile diffraction planes. G200 support presents main diffractions at 2θ = 26.4°, 42.6°, 44.4°, and 54.6°, corresponding to the (002), (004), (100), and (101) planes of graphite [33,34].

Moreover, Figure 1 displays an evident XRD pattern difference between synthesized ReO_x_/g-C_3_N_4_ and g-C_3_N_4_, ReO_x_/ZrO_2_ and ZrO_2_, and ReO_x_/G200 and G200. For all synthetized catalysts, 2 theta values near 16.6°, 25.5°, 27.6°, 34.8°, 41.5°, 51°, corresponding to crystal planes (110), (210), (003), (310), (303), and (330), respectively, can be observed [23,35]. Although the diffractions of the precursor NH_4_ReO_4_ and ReO_3_ appear at similar angles, the absence of any contribution in the N1s region (*videinfra*), on the XPS of the catalysts ReO_x_/ZrO_2_ and ReO_x_/TiO_2,_ points to the presence of ReO_3_. These results agree with previous studies [36]. For ReO_x_/TiO_2_, the diffraction peaks related to ReO_3_ species are barely visible, the main diffraction at 25.5° being probably masked by anatase. This suggests that ReO_x_ sizes are below the equipment detection limit [23,33,34]. Furthermore, ReO_x_/g-C_3_N_4_ and ReO_x_/G200 also displayed weak diffractions at 2θ of 30.4°, 47.2°, 49.2° and 52.2° which could be due to Re_2_O_7_ [37], as also the XPS showed [23]. Only, ReO_x_/ZrO_2_ showed orthorhombic β-ReO_2_ phase near to 37.3° (200) and 53.9° (221) [38,39].

The XRD confirmed that ReO_3_ is the primary crystalline phase of rhenium on these supports, in agreement with previous results by Bassi et al. [23] over ReO_x_ supported on SiO_2_ and ZrO_2_. Mitra et al. [40] emphasized the importance of rhenium reductions in catalytic performance, particularly in reactions like hydrogenation, where lower oxidation states of rhenium can play a crucial role since not all of the rhenium species are catalytically active [36].

The crystal size of ReO_3_ was estimated using the Scherrer equation, and the results are shown in Table 1, except for ReO_x_/TiO_2_, whose diffractions are too weak. The crystal size for ReO_x_ over carbonaceous support is higher for ReO_x_/g-C_3_N_4_ than for ReO_x_/G200. This could be related to the lower BET area of g-C_3_N_4_. On the other hand, ReO_x_/ZrO_2_ showed crystal sizes closer to those of ReO_x_/G200.

**Figure 2:**
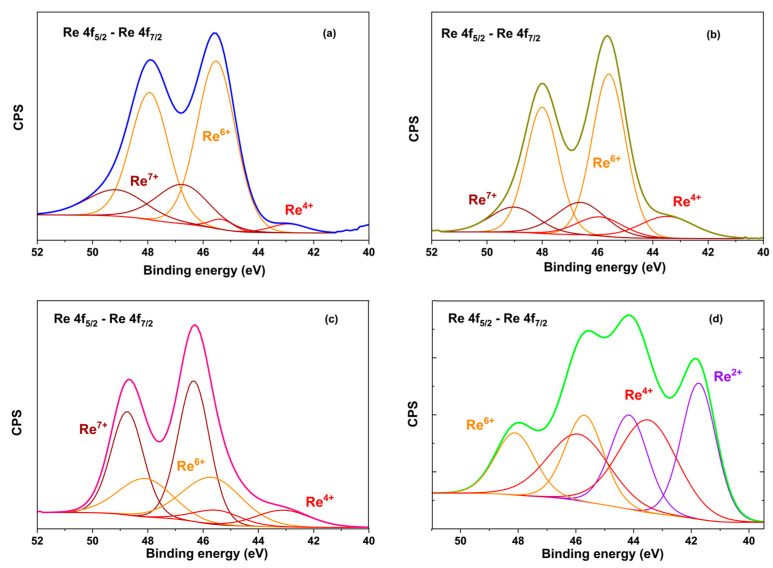
XPS of the Re4f region of fresh ReOx-supported catalysts: (**a**) ReO_x_/G200, (**b**) ReO_x_/g-C_3_N_4,_ (**c**) ReO_x_/ZrO_2,_ and (**d**) ReO_x_/TiO_2_.

XPS was used to evaluate the surface composition of the catalysts. The Zr 3d, Ti 2p, C 1s, and N 1s regions are included in the Supplementary Materials (Figure S1). Figure 2 shows the XPS spectra for the Re 4f core-levels of rhenium oxide-based catalysts characterized by the 4f_5/2_ and 4f_7/2_ orbitals split by 2.4 eV.

The catalysts ReO_x_/g-C_3_N_4_, ReO_x_/G200, and ReO_x_/ZrO_2_ displayed contributions at 46.5–46.7 eV, 45.5–45.7 eV, and 42.9–43.5 eV in the Re 4f 7/2 region, which are ascribed to oxides of Re^+7^, Re^+6,^ and Re^+4^, respectively [41]. In contrast, the XPS spectrum for ReO_x_/TiO_2_ displayed contributions at 41.7, 43.5, and 45.7 eV, which are due to Re^+2^, Re^+4^, and Re^+6^, respectively. This suggests that TiO_2_ promotes reduced rhenium species, in line with the findings of Mitra et al. [40] and Okal et al. [42], who observed similar behavior in rhenium catalysts supported on reducible oxides.

The survey spectrum of the ReO_x_/ZrO_2_ and ReO_x_/TiO_2_ catalysts did not display any contribution in the N1s region, implying that the ammonium salt fully decomposed in the near-surface region during the thermal treatment process. Therefore, the Re^+7^ identified species corresponds to Re_2_O_7_. Table 2 displays BE, Re specie relative atomic percentage, and Re surface atomic ratio for ReOx-supported catalysts in the 4f region.

The area of the different contributions was used to estimate the surface atomic ratios, which can be related to the relative concentration of accessible Re sites [36]. In the present study, this parameter decreases in the following order: ReO_x_/TiO_2_~ReO_x_/ZrO_2_ > ReO_x_/g-C_3_N_4_ > ReO_x_/G200. Therefore, ReO_x_/TiO_2_ and ReO_x_/ZrO_2_ have the highest Re apparent dispersion among the evaluated catalysts in agreement with the XRD patterns. On the contrary, ReO_x_/g-C_3_N_4_ and ReO_x_/G200 showed lower dispersion than the reducible supports, and among them, g-C_3_N_4_ provided better Re dispersion, likely due to the nitrogen functionalities of the support [43].

**Figure 3:**
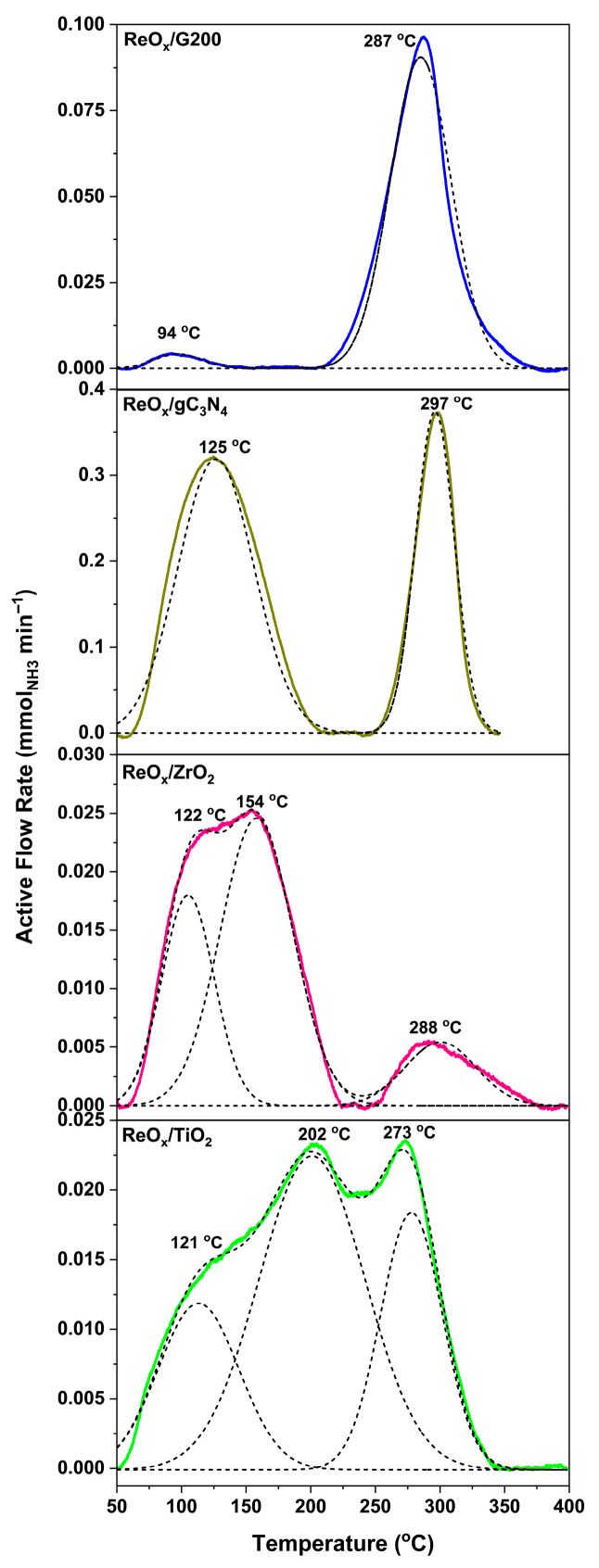
NH_3_ temperature-programmed desorption from ReOx supported catalysts.

Figure 3 presents the NH_3_-TPD of ReO_x_ catalysts. All the samples exhibit desorption signals between 50 and 400 °C, evidencing the presence of different strengths of acid sites. Thus, weak sites desorb below 200 °C, while medium-strong ones do so between 200 and 400 °C [44,45]. ReO_x_/G200 shows two very distinct signals corresponding to weak (94 °C) and strong (287 °C) acidity, the latter being much more intense. ReO_x_/g-C_3_N_4_ also shows two very well-differentiated signals of weak and strong acidity (at 125 °C and 297 °C, respectively), but in this case, the intensity of both signals is much more similar. On the other hand, catalysts with metal oxide supports show three desorption signals. Thus, ReO_x_/ZrO_2_ shows two intense signals centered at 122 and 154 °C and a small one at 288 °C, indicating that weak acid sites dominate with only a minor contribution from medium-strong sites. Similarly, ReO_x_/TiO_2_ displays two desorptions around in the weak acidity region (121 and 202 °C), and a peak at 223 °C related to medium-strong acidity, showing greater relative intensity than that observed in the catalyst supported on ZrO_2_. These results demonstrate that the support nature directly determines both the density and strength of acid sites, in agreement with previous findings [46].

Table 3 shows the weak, medium-strong, and total acidity for each catalyst. The total acidity decreased in the order ReO_x_/g-C_3_N_4_ > ReO_x_/G200 > ReO_x_/TiO_2_ ≈ ReO_x_/ZrO_2_.

It is important to note that the weakest acidity was shown by ReO_x_/ZrO_2_ (88/12) and ReO_x_/g-C_3_N_4_ (63/37). Additionally, ReO_x_/TiO_2_ (25/75) and ReO_x_/G200 (4/96) showed more medium-strong acid sites. The values in brackets correspond to the percentage ratio of weak to medium-strong acid sites derived from the deconvoluted NH_3_-TPD profiles. According to A. Jia et al. [45] and B. Li et al. [47], strong acid sites promote the adsorption of the C=O bond in crotonaldehyde and enhance catalytic activity over Ir/TiO_2_ and Ru/ZnO using H_2_, respectively. However, in the present study, catalysts with high medium-strong acidity showed low crotonaldehyde conversion and crotyl alcohol selectivity. On the other hand, some studies pointed out that weak acid sites promote FA adsorption and decomposition to provide molecular hydrogen. This was corroborated by X. Li et al. [48], who reported that FA decomposition over Au/Ce_x_Zr_1−x_O_2_ catalysts was favored by weak and moderate acidic sites, even without CO formation, and improved the yield of the target product using this acid as a hydrogen donor. Additionally, Z. Yu et al. [49] showed that FA decomposition is preferentially converted to H_2_ and CO_2_ over γ-Mo_2_N supported on nitrogen-doped carbon. This improved selectivity was linked to the higher concentration of weak and moderate acid sites compared to bulk γ-Mo_2_N, which exhibited more strong acid sites and H_2_ selectivity below 1.23% in the whole evaluated temperature range. These two acidity effects may explain the activity and selectivity results obtained in this work. As mentioned above, ReO_x_/ZrO_2_ and ReO_x_/g-C_3_N_4_ catalysts presented a greater weak acidity compared to medium-strong acidity and were also those that showed the best catalytic results, since strong acidity would help the adsorption of crotonaldehyde and high weak acidity would contribute to the decomposition of FA so that it can carry out hydrogenation.

### 2.2. Catalytic Activity

**Figure 4:**
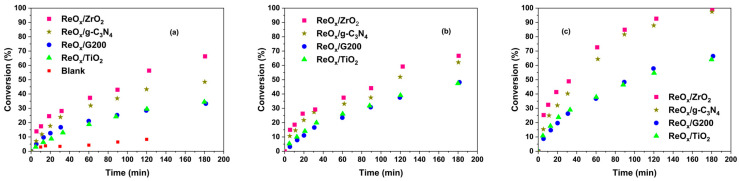
Crotonaldehyde conversion over ReO_x_ catalysts and blank for different reaction temperatures: (**a**) 140 °C, (**b**) 160 °C, and (**c**) 180 °C.

Figure 4 shows crotonaldehyde conversion vs. reaction time of blank and ReO_x_-supported catalysts for all evaluated reaction temperatures, using FA as hydrogen donor. The blank experiment did not show significant crotonaldehyde conversion and selectivity to unsaturated alcohol, confirming that all observed activity arises from the catalytic systems. As shown in Figure 4, all ReO_x_-based catalysts were active within the temperature range of 140–180 °C, and the conversion increased progressively with temperature and reaction time. At 140 °C (Figure 4a and Figures S2–S5), crotonaldehyde conversion remained below 35% (ReO_x_/G200 and ReO_x_/TiO_2_), 50% (ReO_x_/g-C_3_N_4_), and 70% (ReO_x_/ZrO_2_) for all evaluated reaction times. Increasing the temperature to 160 °C (Figure 4b) led to a similar conversion performance to that presented at 140 °C for times less than 60 min and moderate enhancement for longer times. At 180 °C (Figure 4c), the catalytic conversion markedly improved: ReO_x_/ZrO_2_ achieved the highest conversion at 180 min (around 99%), followed by ReO_x_/g-C_3_N_4_ (near 97%), whereas ReO_x_/TiO_2_ and ReO_x_/G200 exhibited lower conversions (≈65%).

**Figure 5:**
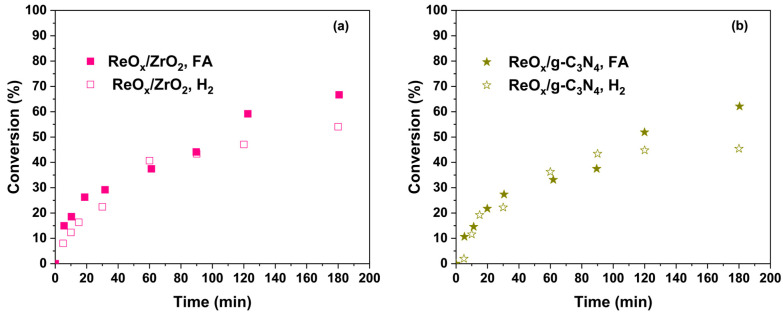
Crotonaldehyde conversion over ReO_x_ catalysts supported on (**a**) ZrO_2_ and (**b**) g-C_3_N_4_ at 160 °C, varying the hydrogen source (FA and H_2_).

Figure 5 shows a comparison between crotonaldehyde conversion for the most active and selective catalysts: ReO_x_/ZrO_2_ (Figure 5a) and ReO_x_/g-C_3_N_4_ (Figure 5b) at 20 bar and 160 °C, varying the hydrogen source. Both catalysts displayed similar conversion profiles under the two conditions for times below 90 min. For ReO_x_/ZrO_2_, conversion reached roughly 66% with FA and 54% with H_2_ after 180 min, while for ReO_x_/g-C_3_N_4_ the final conversions were around 62% for FA and 45%for H_2_. This evidence confirms that FA effectively serves as an in situ hydrogen donor, providing active crotonaldehyde hydrogenation without the need for external H_2_.

**Figure 6:**
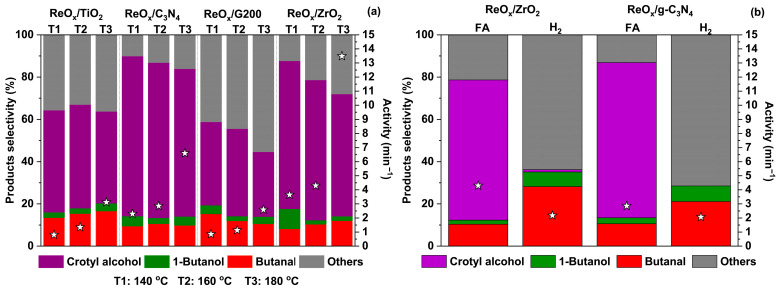
Products selectivity distribution (bars) and catalyst activity (stars), at 25% of crotonaldehyde conversion: (**a**) for all supported ReO_x_ catalysts, using FA as hydrogen donor for all evaluated temperatures, and (**b**) for ReO_x_/g-C_3_N_4_ and ReO_x_/ZrO_2_ at 160 °C, comparing the hydrogen source (H_2_ and FA).

Figure 6 displays the product selectivity distribution and catalytic activity for all prepared catalysts and reaction temperatures at 25% of crotonaldehyde conversion. According to Figure 6a, it can be observed that the main product for the tested ReO_x_ catalysts is crotyl alcohol when FA is used, except for ReO_x_/G200. The most selective catalysts were ReO_x_/g-C_3_N_4_ and ReO_x_/ZrO_2,_ which reached selectivity to crotyl alcohol of 75.7% and 69.9% at 140 °C. Then, ReO_x_/TiO_2_ and ReO_x_/G200 provided a selectivity of 48.2% and 39.4% at 140 °C.

In addition to hydrogenation products, various condensation compounds have been detected, such as butyraldehyde and crotonic acid. They are collectively categorized as “Others”. These condensation products could be directly catalyzed by the large amount of H^+^ ionized from water and FA [50]. Finally, 1-butanol was the product with the lowest selectivity for all catalysts.

ReO_x_/ZrO_2_ and ReO_x_/g-C_3_N_4_ are more selective for C=O hydrogenation than ReO_x_/G200 and ReO_x_/TiO_2_. The effect of nitrogen atoms on the adsorption of carbonyl groups has been previously studied. For instance, in the hydrogenation of cinnamaldehyde, improved selectivity towards cinnamyl alcohol was observed when nitrogen was incorporated into carbon-based supports [51]. This effect appears to be related to the interaction between C=O groups and nitrogen atoms, which favors the cleavage of the C-O bond. In this context, molybdenum nitride has also been investigated as a catalyst in the hydrogenation of crotonaldehyde, yielding good selectivity results in the liquid phase [52].

On the other hand, most of the catalysts have a decrease in unsaturated alcohol selectivity with an increase in reaction temperature. Crotyl alcohol selectivity decreased by 2.9–5.2% when increasing the reaction temperature from 140 to 160 °C, and by 4.9–25.7% from 160 to 180 °C. Among all catalysts, only ReO_x_/TiO_2_ maintained a relatively stable selectivity across the evaluated temperature range. The overall increase in selectivity toward other products with rising temperature occurred at the expense of crotyl alcohol formation. Additionally, catalysts supported on reducible oxides exhibited a notable increase in butanal selectivity, likely due to the preferential adsorption of the C=C bond on active sites [21]. According to Mironenko et al. [53], the aqueous-phase hydrogenation of furfural at 50 or 100 °C and a total pressure of 3.0 MPa mainly showed unsaturated alcohol, while selectivity depended on the used catalyst. At 150 or 200 °C, these authors observed that the products of furfural catalytic transformations were mainly cyclopentanone, 4-oxo-pentanal, and 5-hydroxypentan-2-one.

Figure 6a displayed that catalyst activity was more pronounced at 180 °C, and for all reactions, temperatures followed the decreasing catalyst order ReO_x_/ZrO_2_ > ReO_x_/g-C_3_N_4_ > ReO_x_/TiO_2_ > ReO_x_/G200. Furthermore, Figure 6b shows that the use of FA as a hydrogen donor led to higher activities compared to the use of H_2_. In addition, it shows that the production of crotyl alcohol was very low or did not exist when H_2_ was used, and the most prominent selectivity was “Others”. According to Naharro et al. [21], gas-phase crotonaldehyde hydrogenation using H_2_ instead of FA as a hydrogen source showed that butanal and butanol were the main products. Therefore, it was proved that the change in the supports and hydrogenating agents would have a high incidence on crotonaldehyde conversion and crotyl alcohol selectivity. In addition, it corroborated that the use of FA as a hydrogen donor allowed for achieving higher unsaturated alcohol selectivity (Figure 6b). On the other hand, Lan and Wang [14] reported catalyst activity of crotonaldehyde hydrogenation on the liquid phase and using H_2_O as solvent (T: 30 °C, P_H_2__: 8 bar) for Ir-ReO_x_/SiO_2_ and Ir-MoOx/SiO_2_. The values were 1.1 and 2.0 min^−1^, respectively. Although this last reference develops a reaction in mild conditions and uses H_2_, this work showed that ReO_x_/TiO_2_ and ReO_x_/G200 had similar activities at 140 and 160 °C, with those reported by the mentioned reference. At the same temperatures, ReO_x_/ZrO_2_ and ReO_x_/g-C_3_N_4_ showed activities above reference values. On the other hand, at 180 °C, the activity for all catalysts was above the reported values. Several works in crotonaldehyde hydrogenation report the sacrifice of activity to obtain high selectivity [5]. The same trend is observed in this work with the increase in temperature.

**Figure 7:**
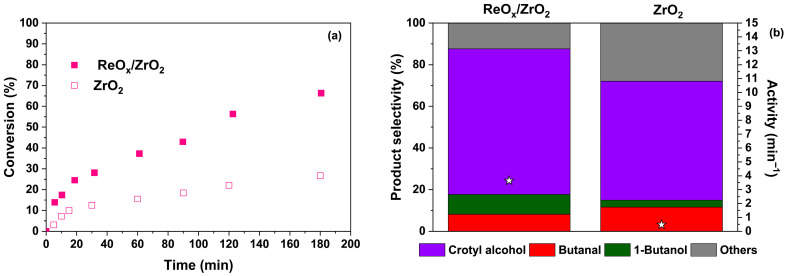
Crotonaldehyde conversion at 140 °C (**a**) and product selectivity (bars) and activity (stars) at 25% of conversion (**b**) for ReO_x_/ZrO_2_ and ZrO_2_.

Furthermore, to evaluate the effect of rhenium impregnation on support, Figure 7 displays the comparison of crotonaldehyde conversion, product selectivity, and catalyst activity for ReO_x_/ZrO_2_ and ZrO_2_ at 140 °C, using FA as hydrogen donor.

According to Figure 7, the conversion and catalyst activity are harnessed by rhenium impregnation over reducible support (ZrO_2_). The crotonaldehyde conversion behavior over time of ZrO_2_ is like ReO_x_/TiO_2_ and ReO_x_/G200 at 140 °C (Figure 4a). Moreover, the selectivity of crotyl alcohol and catalyst activity is improved by ReO_x_ species addition (Figure 7b). Therefore, rhenium oxides over ZrO_2_ and the use of FA are a good option for the catalytic transfer hydrogenation (CTH) process in crotonaldehyde hydrogenation and the C=O bond selectivity to obtain crotyl alcohol. Additionally, it has been reported that ZrO_2_ is a good material for FA decomposition. However, H_2_ selectivity depends on the synthesis preparation method and reaction conditions [54].

The hydrogenation of crotonaldehyde can follow two distinct reaction pathways. Selective hydrogenation of the carbonyl (C=O) bond results in the formation of crotyl alcohol, whereas hydrogenation of the C=C bond leads to butanal. Subsequent hydrogenation of the remaining functional group, whether C=C or C=O, ultimately yields butanol as the fully hydrogenated product [6]. Low conversion levels may be associated with the strong and competitive adsorption of C=O groups from both FA and crotonaldehyde on the active sites [21]. In this work, a clear dependence of conversion and selectivity on the nature of the support was observed, mainly attributed to the ratio of weak and medium-strength acid sites. Furthermore, support also influences the distribution of rhenium species in the catalysts, an important factor influencing their performance. ReOx catalysts supported on ZrO_2_ and g-C_3_N_4_ appear to mitigate this adsorption competition, potentially by generating new or more accessible active sites through specific interactions between ReO_x_ species and the support surface. On the other hand, these supports may facilitate FA decomposition without interfering with the active sites responsible for crotonaldehyde adsorption, while simultaneously promoting selective activation of the C=O bond. This is supported by the results shown in Figure 7, where crotonaldehyde conversion and crotyl alcohol selectivity are reported using FA as a hydrogen donor and bare ZrO_2_ (without ReO_x_ species) as the heterogeneous catalyst.

Figures S2–S5 present the evolution of crotonaldehyde conversion and product selectivity over time for all evaluated catalysts, following the graphical structure reported by S. Ojwach et al. [55]. As shown in the Supplementary Materials, crotonaldehyde conversion increases with time, while product selectivity varies depending on the reaction time, temperature, and used catalyst. Most of the catalysts showed that crotyl alcohol selectivity tends to a maximum and stable value at higher reaction time.

### 2.3. Kinetic Analysis

Table 4 shows the kinetic parameters for all prepared catalysts. These parameters are activation energy, pre-exponential factor, and first-order kinetic constant for each evaluated reaction temperature. The E_a_ of supported carbonaceous catalysts is lower than for metal oxide-supported catalysts. Kinetic constants increase with temperature, as expected.

According to Table 4, ReO_x_/G200 has the lowest Ea. Nevertheless, its kinetic constants present lower values, if compared with ReO_x_/ZrO_2_. Therefore, a preexponential factor (A) has a high incidence in catalytic crotonaldehyde conversion, and, therefore, a catalyst with the lower Ea is not the best for crotonaldehyde conversion and crotyl alcohol selectivity. Moreover, the initial kinetic rate was calculated for all reaction temperatures and ReO_x_ catalysts. The results are shown in Table 5.

The initial reaction rates increase with temperature. Nevertheless, this kinetic parameter does not change significantly between 140 and 160 °C. The maximum initial rate value for all catalysts was at 180 °C. According to the initial rate, the order of this parameter is: ReO_x_/ZrO_2_, ReO_x_/g-C_3_N_4_, ReO_x_/TiO_2,_ and ReO_x_/G200. On the other hand, Table 6 shows intrinsic kinetic rates at reaction temperatures for all evaluated catalysts.

The intrinsic kinetic rate is enhanced by increasing temperature for all catalysts. The intrinsic kinetic rate of catalysts follows the trend: ReO_x_/g-C_3_N_4_ > ReO_x_/G200 > ReO_x_/ZrO_2_ > ReO_x_/TiO_2_. Although/ReO_x_/g-C_3_N_4_ shows a better intrinsic kinetic rate, this catalyst presents a lower crotonaldehyde conversion than ZrO_2_ for all evaluated reaction temperatures. This behavior could be explained by the differences in the surface area and nature of the supports [23,25,36]. On the other hand, a fraction of the exposed Re species on TiO_2_ and on G200 constitutes inactive Re species for crotonaldehyde conversion, leading to the lower activity of ReO_x_/TiO_2_ and ReO_x_/G200 catalysts compared to ReO_x_/g-C_3_N_4_ and ReO_x_/ZrO_2_ [36].

### 2.4. Reaction Mechanism over ReO_x_ Supported Catalyst

The efficiency of heterogeneous catalytic processes, including both activity and selectivity, is largely determined by the adsorption, activation, and mechanism of interaction of reactants on the catalyst surface [45,56]. Based on experimental data and literature evidence, crotonaldehyde hydrogenation mechanisms are proposed, both using FA as a hydrogen donor and directly H_2_. In these mechanisms, only α,β-unsaturated aldehyde adsorption to produce the unsaturated alcohol was considered, in the same way that previous works [6,8,10,56].

**Figure 8:**
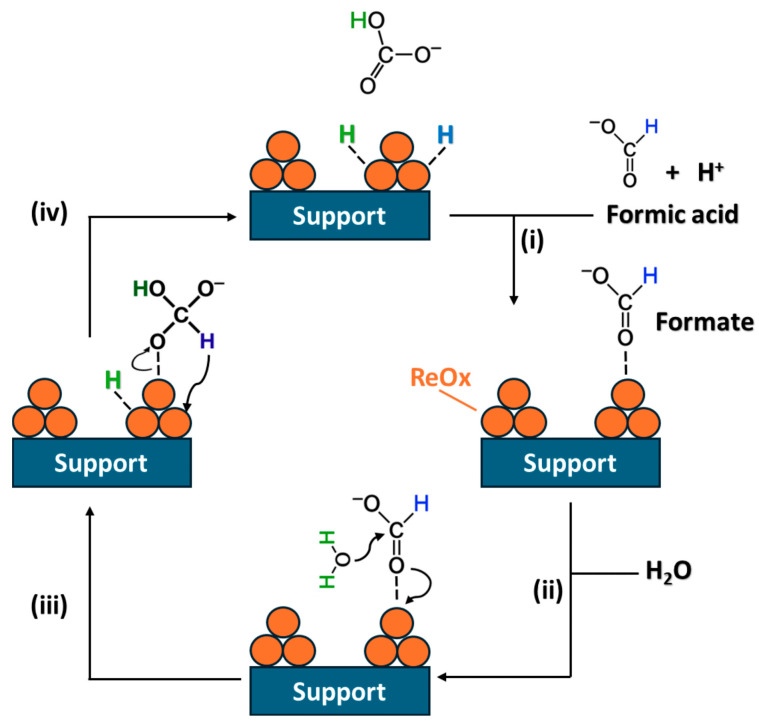
Proposed reaction mechanism for FA activation by formate dehydrogenation with H_2_O over ReO_x_ supported catalysts. (i) formate adsorption; (ii) water attack; (iii) cleavage of the C–H bond and (iv) bicarbonate ion desorption.

The coexistence of the crotonaldehyde and hydrogen donors can induce competitive adsorption between them on the catalyst surface, thus leading to different dehydrogenation degrees of the hydrogen donor [56]. Therefore, the mechanism discussion starts with the activation of FA on the catalyst surface in the absence of crotonaldehyde. There are several routes of the FA activation mechanism: FA dehydrogenation, FA dehydration, and the dehydrogenation of formate water-involved route [57,58,59,60]. Due to in this work, water is used as a solvent, dehydrogenation of formate is the appropriate mechanism to consider for FA activation [56,58,59]. According to Nie et al. [61], several studies report that formic acid ionizes in aqueous media, and water acts as a proton donor, facilitating the formation of surface H^+^ species that participate in the hydrogenation pathway. The water-involved formate dehydrogenation was proposed as follows in Figure 8.

Formate dehydrogenation was proposed to proceed via the following sequence of steps: (i) the formate ion adsorbs linearly onto ReO_x_ species through coordination of the carbonyl oxygen lone pair; (ii) a water molecule attacks the carbon center of the adsorbed formate, generating a carbonic acid as an intermediate and releasing a surface-bound hydrogen atom; (iii) subsequent cleavage of the C–H bond in this intermediate produces an additional hydrogen atom adsorbed on the surface; (iv) two H atoms on the catalyst surface remain adsorbed and bicarbonate ion molecule is desorbed. Isotope-labeling experiments with deuterated water (D_2_O) and deuterated sodium formate (DCOONa) provide strong evidence that the water insertion step into the adsorbed formate species constitutes the rate-determining step of the reaction [56].

**Figure 9:**
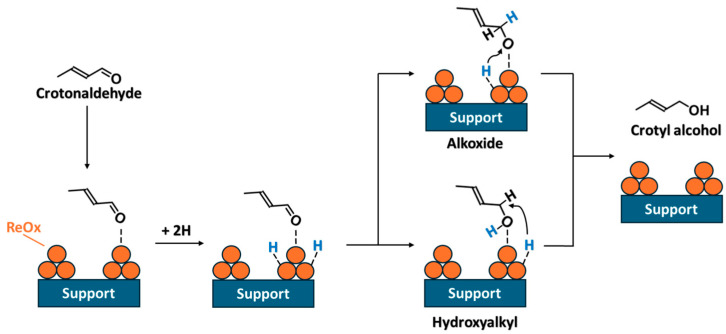
Proposed reaction mechanism of crotonaldehyde activation on the ReO_x_-based catalyst surface.

To continue with the entire process, Figure 9 shows the reaction mechanism of crotonaldehyde activation on the ReO_x_-based catalyst surface with weak affinity for C=C bonds in the presence of surface hydrogen atoms.

First, a crotonaldehyde molecule is adsorbed on the ReO_x_ species over the catalyst surface. Then, the surface H atom derived from the hydrogen donor attacks the carbonyl group of crotonaldehyde, leading to the formation of either an alkoxide intermediate, through bonding to the oxygen atom, or a hydroxyalkyl intermediate, through bonding to the carbon atom, which subsequently hydrogenates crotonaldehyde to crotyl alcohol. Computational studies indicate that the pathway proceeding via the hydroxyalkyl intermediate, where the surface hydrogen initially attacks the carbonyl oxygen of adsorbed α,β-unsaturated aldehyde, requires a lower activation energy than the route involving the alkoxide intermediate [62].

According to the characterization and catalytic test results, medium-strong acid properties of the catalysts play a crucial role in the adsorption of the C=O bond in crotonaldehyde and subsequent crotyl alcohol selectivity [45]. Additionally, weak acid sites may promote hydrogen source production from FA decomposition, as described previously [48,49].

**Figure 10:**
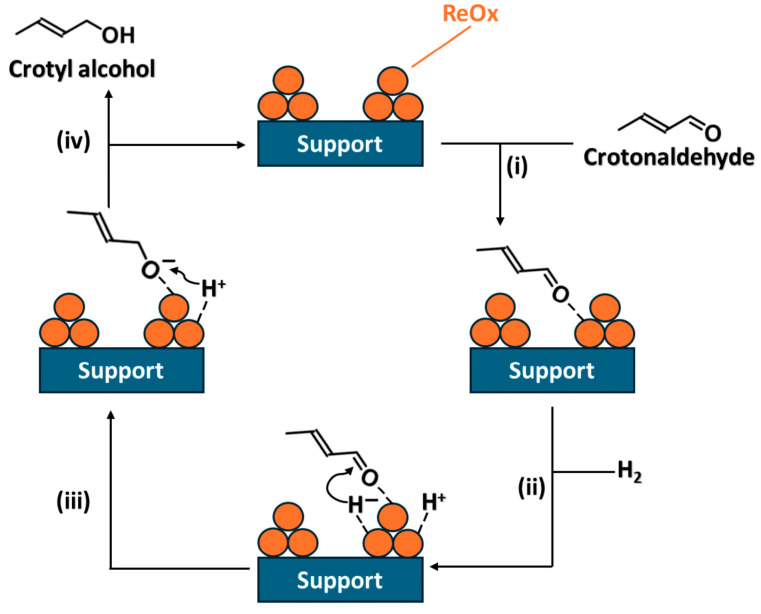
Proposed reaction mechanism of crotonaldehyde hydrogenation to crotyl alcohol using H_2_ and over ReO_x_ supported catalysts. (i) crotonaldehyde adsorption; (ii) hydrogen heterolytic dissociation; (iii) hydride species attack and (iv) protonation of alkoxide intermediate.

On the other hand, the plausible reaction mechanism for crotonaldehyde hydrogenation to crotyl alcohol is proposed in Figure 10, using H_2_ and over ReO_x_-supported catalysts.

This reaction mechanism proceeds in four main steps [6,8]: (i) Crotonaldehyde adsorbs onto the catalyst surface via the aldehyde group, which is subsequently activated by ReO_x_. Then, (ii) hydrogen is heterolytically dissociated at the ReO_x_-support interface to generate hydride (H^−^) and proton (H^+^) species. The hydride species attack the carbonyl carbon of the adsorbed crotonaldehyde (iii), forming an alkoxide intermediate. Ionic hydrogenation with hydride (H^−^) species is generally effective for the hydrogenation of polar double bonds in organic synthesis [63,64,65]. Additionally, the notably high selectivity to crotyl alcohol observed over some of the ReO_x_-supported catalysts can be mainly attributed to the generation of H^+^ and H^−^ species on the catalyst surface [6]. However, low H_2_ solubility in water could be unfavorable to produce enough hydrogen ionic species [56]. Finally, (iv) the alkoxide intermediate is protonated to yield crotyl alcohol. The rate-determining step is the hydride attack on the carbonyl carbon (step iii) [6,8,65]. The high selectivity arises from the generation of hydride species at the ReO_x_-support interface and activation of the aldehyde group on ReO_x_, enabling ionic hydrogenation. On the other hand, high catalytic activity is attributed to efficient aldehyde activation and the proximity of crotonaldehyde to active hydride species due to adsorption on ReO_x_.

In this work, when molecular hydrogen is used, butanal and butanol were the main products, and crotyl alcohol was too low or absent. Therefore, the H_2_ cleavage is achieved into H^+^ and H^−^ species by ReO_x_/ZrO_2_ and ReO_x_/g-C_3_N_4_. Nevertheless, these ionic species attack preferably the C=C bond on crotonaldehyde to produce butanal or favor the over-hydrogenation of unsaturated bonds to produce 1-butanol derived from butanal or crotyl alcohol.

Currently, the detailed structure of the evaluated catalysts remains unclear. Future work will focus on advanced characterization to elucidate the role of rhenium oxides and the structural features of the catalysts.

## 3. Materials and Methods

### 3.1. Catalysts’ Synthesis

**Figure 11:**
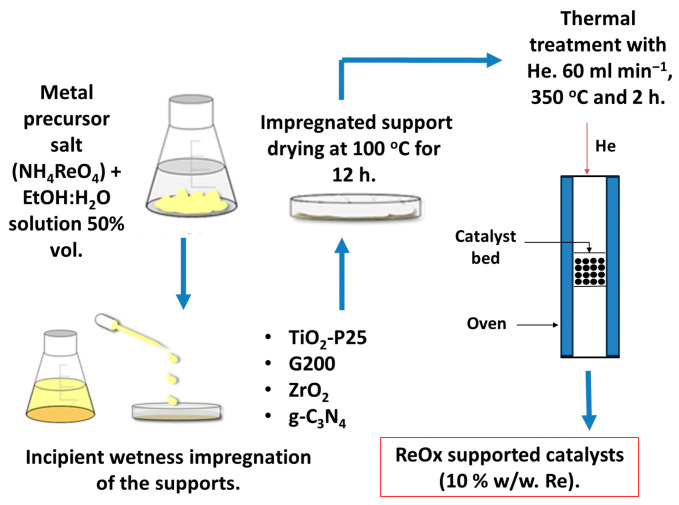
Flow diagram for ReO_x_-supported catalysts synthesis.

The ZrO_2_ support was prepared in the laboratory through precipitation, using ZrO(NO_3_)_2_·xH_2_O as a precursor salt and calcined at 500 °C for 3 h. The g-C_3_N_4_ support was obtained by thermal treatment of urea at 450 °C for 4 h in a muffle furnace inside a closed recipient at a heating rate of 15 °C min^−1^. Additionally, two commercial supports were considered: G200 (High surface area graphite, Timcal, Bodio, Switzerland) and TiO_2_-P25. Then, rhenium catalysts were prepared by incipient wetness impregnation to achieve a 10 wt%. loading of this metal on the supports. The precursor salt used was NH_4_ReO_4_ (Sigma-Aldrich, St. Louis, MO, USA. ≥99%), which was dissolved in a 50/50 EtOH/H_2_O (% vol.) solution. Following the impregnation step, the catalysts were dried at 100 °C for 12 h. Finally, they were treated with He (60 mL min^−1^) for 2 h at 350 °C to obtain ReO_x_ on the supports. The support selection is based on previous works that report differences in acid-base properties and acid medium stability on catalytic reactions [66,67,68,69,70,71,72]. ZrO_2_ and TiO_2_ were included as reducible oxides with different metal–support interactions, while g-C_3_N_4_ and G200 were chosen as carbon-based materials with contrasting surface nature. This diversity seeks to compare and correlate the physicochemical properties of the supports with catalytic activity and selectivity. Figure 11 shows the flow diagram for catalysts synthesis. It describes all synthesis stages from incipient wetness impregnation to thermal treatment with He.

### 3.2. Catalyst Characterization

#### 3.2.1. Surface Area and Textural Properties

Apparent surface area (m^2^ g^−1^) of ZrO_2_, TiO_2_, g-C_3_N_4,_ and all ReO_x_ supported catalysts were obtained from N_2_ adsorption isotherms at 77 K. These measurements were developed using a Micromeritics ASAP-2000 automatic apparatus (Norcross, GA, USA).

#### 3.2.2. X-Ray Diffraction (XRD)

XRD patterns of the samples were recorded on a Polycristal X′ Pert Pro PANalytical diffractometer (Malvern, UK) via Ni-filtered Cu Kα radiation (λ = 1.54 Å). All the supports and catalysts were analyzed under the conditions of 45 kV and 40 mA, using a 2θ range from 4° to 85° and a step of 0.04° s^−1^. The Scherrer equation is used to calculate the particle sizes in the catalyst sample [25,28,73]. It is represented by Equation (1).

(1) $$\beta \left(2\theta \right)=\frac{K\cdot \lambda }{L\cdot cos(2\theta )}$$

where β(2θ) is the peak width in radians at the value of 2θ. θ is the diffraction angle in radians, λ is the X-ray wavelength (1.5406 Å or 0.15406 nm), and L is the crystallite size (nm). The constant K is a function of the crystallite shape but is generally approximated as 1.0 for spherical particles [73]. Nevertheless, there are reports that set K constant at 0.9 [28,74]. In this study, K is set as 0.9, and the most intense peaks are used to select 2θ.

#### 3.2.3. X-Ray Photoelectron Spectroscopy (XPS)

The XPS spectra were obtained by using non-monochromatic Al radiation (200 W, 1486.61 eV) through SPECS GmbH (Specs Group, Berlin, Germany) with a UHV system and with an energy analyzer PHOIBOS 150 9MCD (Specs Group, Berlin, Germany). The survey spectra were obtained with a 50 eV pass energy, and region spectra were obtained at 20 eV pass energy. The binding energy (BE) was calibrated taking as a reference the C 1s peak of graphitic carbon at 284.6 eV, and the equipment error was considered as less than ±0.01 eV for the determination of energies.

The Re/Support ratio was determined using XPS by integrating the areas under the peaks corresponding to rhenium and the support element on the spectra (e.g., Ti 2p for TiO_2_ or Zr 3d for ZrO_2_). Each peak area was corrected by applying the appropriate relative sensitivity factor (RSF) to account for the differing photoemission cross-sections.

#### 3.2.4. Ammonia Temperature-Programmed Desorption (NH_3_-TPD)

Initially, the samples were pretreated at 350 °C for 30 min with a He flow of 25 mL min^−1^. Then, the system was cooled to 50 °C and the sample was ammonia saturated at this same temperature for 60 min using a 25 mL min^−1^ flow of NH_3_ (5% vol.). After adsorption, physically adsorbed ammonia was removed by purging the system with He for 30 min. Subsequently, desorption was performed under He flow (25 mL min^−1^) with a ramp of 10 °C min^−1^ up to 400 °C. The desorbed species were monitored using a mass spectrometer by tracking the *m*/*z* = 17 fragment corresponding to NH_3_.

#### 3.2.5. Catalytic Test

**Figure 12:**
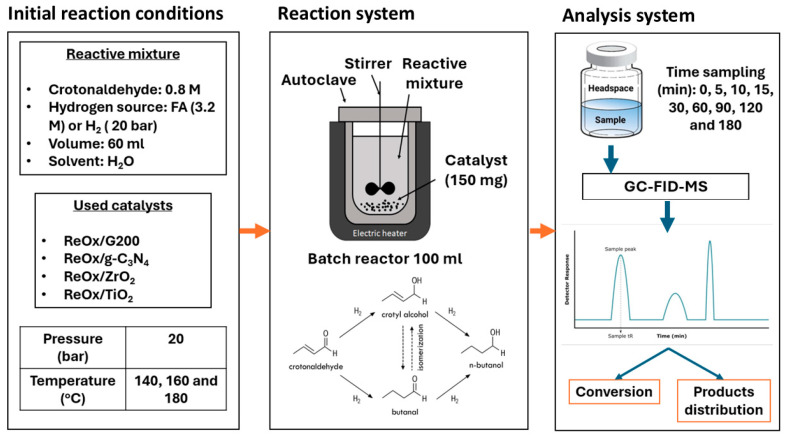
Catalytic test scheme, including initial reaction conditions, reaction system and analysis system.

The hydrogenation experiments were conducted in a 100 mL batch reactor from Parr Instruments (St, Moline, IL, USA), operated in discontinuous mode. The reactive mixture was an aqueous solution of 60 mL, 0.4 M crotonaldehyde (Sigma-Aldrich, ≥99%) and 3.2 M FA (Sigma-Aldrich, ≥96%). Once the reactive mixture was introduced into the reactor with 150 mg of catalyst (reactant-to-catalyst molar ratio of 315), the reactor was purged three times with N_2_, then pressurized to 20 bar with the same inert gas. The reaction temperatures studied were 140, 160, and 180 °C. Additionally, a blank test without catalyst and ZrO_2_ (150 mg) was performed at 140 °C with the same initial conditions described above. On the other hand, to probe the incidence of hydrogenating agent, H_2_ was employed at 20 bar and 160 °C over ReO_x_/ZrO_2_ and ReO_x_/g-C_3_N_4_. In this case, 200 mg of catalyst was used, and the reactive solution was 80 mL with the same crotonaldehyde concentration and water as solvent. For each experiment, small aliquots of the reaction mixture were collected periodically, and the samples were analyzed using an Agilent 8860 GC-FID (Agilent, Santa Clara, CA, USA) with a mass detector to measure and identify the concentrations of reactants and reaction products. Figure 12 shows the flow diagram for catalytic tests, including initial reaction conditions, reaction system, and analysis system.

Equation (2) is used to determine the conversion of crotonaldehyde, while Equation (3) is used to calculate the selectivity for each reaction product. Additionally, catalytic activity is considered using Equation (4) at a conversion of 25%.

(2) $$X_{croton}=\frac{C_{Croton}^{o}-C_{Croton}^{t}}{C_{Croton}^{o}}\cdot 100\%$$

(3) $$S_{i}=\frac{\#C_{i}\cdot C_{i}^{t}}{\sum_{1}^{j}{\#C}_{j}\cdot C_{j}^{t}}\cdot 100\%$$

(4) $$a=\frac{∆n_{Croton}}{∆t\cdot n_{Re}}$$

In Equation (2), X_Croton_ represents the conversion percentage (%) of crotonaldehyde, where $C_{Croton}^{o}$ and $C_{Croton}^{t}$ denote the initial concentration and the concentration at time (t) of this α, β-unsaturated aldehyde, respectively, in molarity (M). Meanwhile, in Equation (3), S_i_ indicates the selectivity of component i in percentage (%), #C_i_ is the number of carbon atoms present in molecule i, $C_{i}^{t}$ is the concentration in molarity (M) of component i at time t, and $\sum_{1}^{j}{\#C}_{j}*C_{j}^{t}$ is the summation of the product of carbon atoms and the concentrations (M) for each reaction product at time t. In Equation (4), a denotes the catalytic activity in min^−1^, Δn_Croton_ is the moles of crotonaldehyde converted, Δt is the required time for the specified conversion of crotonaldehyde (in minutes), and n_Re_ is the moles of rhenium present in the amount of catalyst used for the reaction.

#### 3.2.6. Kinetic Evaluation

To further analyze insight into the CTH of crotonaldehyde to crotyl alcohol over the ReO_x_ supported catalyst, its kinetics were investigated at three different reaction temperatures (140, 160, and 180 °C), assuming that the conversion of crotonaldehyde follows first-order reaction kinetics (Equation (5)), as it is proposed by He et al. [11] and Ren et al. [15] for furfural hydrogenation to obtain furfuryl alcohol with the CTH process. Additionally, the assumption is feasible since FA is added in excess ([FA] = 8∙[Crotonaldehyde]).

(5) $${-r}_{CRO}=k\cdot C_{CRO}$$

where −r_CRO_ is the rate of consumption of crotonaldehyde (mol L^−1^ min^−1^), C_CRO_ is the crotonaldehyde concentration (M), and k is the rate constant (min^−1^). To calculate the activation energy (E_a_) and pre-exponential factor (A), the following expression is used: k = A·exp(−Ea·(RT)^−1^). The initial reaction rate (mol min^−1^ g_cat_^−1^) is calculated from the initial slope of the conversion vs. time plot, according to Equation (6). The same math expression is used by Leiva et al. [75] and Díaz et al. [33] to determine this parameter.

(6) $${-r}_{CRO}^{o}=\frac{{n\cdot b}_{CRO}}{g_{cat}}$$

To assess the intrinsic rate, the initial reaction rates are normalized by the exposed Re surface species, determined from the XPS surface atomic ratio [25]. The intrinsic rate is expressed as the number of crotonaldehyde molecules converted per exposed surface Re atom per minute (molecules_CRO_ atoms Re^−1^ min^−1^), is calculated by Equation (7).

(7) $$r_{i}=\frac{r_{s}\cdot {MW}_{support}}{\left(1-{Wt}_{Re}\right)\cdot (Re/M{)}_{XPS}}$$

where r_i_ is the intrinsic rate, r_s_ is the initial reaction rate (mol_Cro_ g^−1^ min^−1^), MW_Support_ is the molar mass of the support, Wt_Re_ is the Re loading (wt%), and (Re/M)_XPS_ is the XPS surface atomic Re/Support ratio (atom atom^−1^).

## 4. Conclusions

Catalytic hydrogenation of crotonaldehyde was effectively accomplished using ReO_x_ catalysts supported on G200, g-C_3_N_4_, TiO_2_, and ZrO_2_, employing FA as an in situ hydrogen donor. The catalytic performance significantly depended on the nature of the support and reaction temperature. Increasing the reaction temperature (140–180 °C) enhanced crotonaldehyde conversion but negatively impacted selectivity toward crotyl alcohol.

Among the tested catalysts, ReO_x_/ZrO_2_ and ReO_x_/g-C_3_N_4_ demonstrated superior catalytic activities and higher selectivity toward crotyl alcohol, emphasizing the critical role of the support’s physicochemical properties in determining catalyst performance. FA proved to be a more effective hydrogen donor compared to molecular hydrogen (H_2_, 20 bar), yielding improved crotyl alcohol production.

Characterization studies confirmed the presence of distinct Re species influenced by support. Carbonaceous supports predominantly stabilized higher oxidation states of Re (mainly Re^6+^ and Re^7+^), whereas TiO_2_ favored the formation of more reduced species. The ZrO_2_-based catalyst presented the highest proportion of Re^7+^. Additionally, carbonaceous materials exhibited greater susceptibility to pore blockage after Re impregnation and thermal treatment, unlike reducible oxide supports, which maintained their textural properties. Additionally, catalysts with more weak acid sites showed the best activity and crotyl alcohol selectivity, using FA as hydrogen donor.

Overall, this study underlines that selecting appropriate supports and hydrogen-donor molecules significantly influences catalytic efficiency, offering valuable insights for the optimization of ReO_x_-based catalytic systems toward the sustainable production of crotyl alcohol from crotonaldehyde.

## Figures and Tables

**Table 1:** Textural characteristics of supports and synthesized ReO_x_ catalysts.

Material	BET Area (m^2^ g^−1^)	ReO_3_ Crystal Size ^1^ (nm)
TiO_2_	53	-
ZrO_2_	52	-
g-C_3_N_4_	202	
G200	200	-
ReO_x_/TiO_2_	49	-
ReO_x_/ZrO_2_	52	21
ReO_x_/g-C_3_N_4_	34	35
ReO_x_/G200	96	22

^1^ Calculated using 2θ ≈ 16.6°.

**Table 2:** BE (eV), rhenium specie relative atomic percentage, and rhenium surface atomic ratio for ReO_x_ supported catalysts in the 4f region.

Catalyst	Re 4f_7/2_ BE, eV (%)				Re/X
	Re^2+^	Re^4+^	Re^6+^	Re^7+^	
ReO_x_/ZrO_2_	-	43.5 (11.5)	45.7 (38.7)	46.5 (49.8)	0.38 ^1^
ReO_x_/TiO_2_	41.7 (35.1)	43.5 (40.4)	45.7 (24.5)	-	0.38 ^2^
ReO_x_/g-C_3_N_4_	-	43.5 (14.5)	45.6 (66.2)	46.6 (19.3)	0.027 ^3^ 0.026 ^4^
ReO_x_/G200	-	42.9 (3.6)	45.5 (73.0)	46.7 (23.3)	0.007 ^3^

^1^ X: Zr, ^2^ X: Ti, ^3^ X: C, and ^4^ X: N.

**Table 3:** Surface acid sites of different ReO_x_ catalysts.

Catalyst	Acidity (µmol NH_3_ g_cat_^−1^)		
	Weak	Medium-Strong	Total
ReO_x_/G200	24	649	673
ReO_x_/g-C_3_N_4_	483	289	772
ReO_x_/ZrO_2_	303	43	346
ReO_x_/TiO_2_	89	264	353

**Table 4:** Kinetic parameters for ReO_x_ catalysts in crotonaldehyde hydrogenation, using FA as in situ hydrogen donor.

Catalyst	Ea (kJ mol^−1^)	A (min^−1^)	k_140_ (min^−1^)	k_160_ (min^−1^)	k_180_ (min^−1^)
ReO_x_/ZrO_2_	38.06	925.75	0.01427	0.01545	0.0385
ReO_x_/g-C_3_N_4_	41.50	279.08	0.00866	0.00994	0.0255
ReO_x_/TiO_2_	50.02	7102.48	0.00339	0.00648	0.0123
ReO_x_/G200	23.11	4.40	0.00580	0.00588	0.0106

**Table 5:** Initial kinetic rates (mol min^−1^ g_cat_^−1^) for ReO_x_ supported catalysts.

Catalyst	140 °C	160 °C	180 °C
ReO_x_/ZrO_2_	0.395	0.423	1.020
ReO_x_/TiO_2_	0.122	0.185	0.341
ReO_x_/g-C_3_N_4_	0.241	0.269	0.708
ReO_x_/G200	0.169	0.172	0.304

**Table 6:** Intrinsic kinetic rate (molecules_croton_ min^−1^ atoms_Re_^−1^) for ReO_x_ supported catalysts at evaluated reaction temperatures.

Catalyst	140 °C	160 °C	180 °C
ReO_x_/ZrO_2_ ^1^	142.57	152.54	367.59
ReO_x_/TiO_2_ ^2^	28.39	43.25	79.71
ReO_x_/g-C_3_N_4_ ^3^	912.84	1020.03	2682.16
ReO_x_/G200 ^3^	308.85	314.74	555.05

^1^ X: Zr, ^2^ X: Ti, and ^3^ X: C.

## Data Availability

The raw data supporting the conclusions of this article will be made available by the authors on request.

## Supplementary Materials

The following supporting information can be downloaded at: https://www.mdpi.com/article/10.3390/molecules30214307/s1, Figure S1: XPS regions for, ReO_x_/TiO_2_ (a) Ti 2p, ReO_x_/ZrO_2_: (a) Zr 3d, ReO_x_/G200 (c) C 1s and ReO_x_/g-C_3_N_4_ (d) C 1s and (e) N 1s; Figure S2: Crotonaldehyde conversion and products selectivity over time for ReO_x_/ZrO_2_ and ZrO_2_; Figure S3: Crotonaldehyde conversion and products selectivity over time for ReO_x_/g-C_3_N_4_; Figure S4: Crotonaldehyde conversion and products selectivity evolution with time for ReO_x_/TiO_2_; Figure S5: Crotonaldehyde conversion and products selectivity over time for ReO_x_/G200; Figure S6: XPS survey spectrums for: (a) ReO_x_/ZrO_2_, (b) ReO_x_/TiO_2_, (c) ReO_x_/g-C_3_N_4,_ and (d) ReO_x_/G200; Table S1: XPS parameters for all ReO_x_-supported catalysts.

## Abbreviations

The following abbreviations are used in this manuscript:

BET area	Brunauer–Emmett–Teller area
CTH	Catalytic transfer hydrogenation
FA	Formic acid
NH_3_-TPD	Ammonia temperature-programmed desorption
RSF	Relative sensitivity factor
XPS	X-ray photoelectron spectroscopy
XRD	X-ray diffraction
