# Supplementary material for: Liquid Phase Catalytic Transfer Hydrogenation of Crotonaldehyde over ReOx-Supported Catalysts Using Formic Acid as In Situ Hydrogen Donor
Source: Molecules. 2025 Nov 5;30(21):4307. doi: 10.3390/molecules30214307 (PMC12610096; doi:10.3390/molecules30214307)
Supplement: Supplementary file 1 [file molecules-30-04307-s001.zip › molecules-3935758-supplementary.pdf]

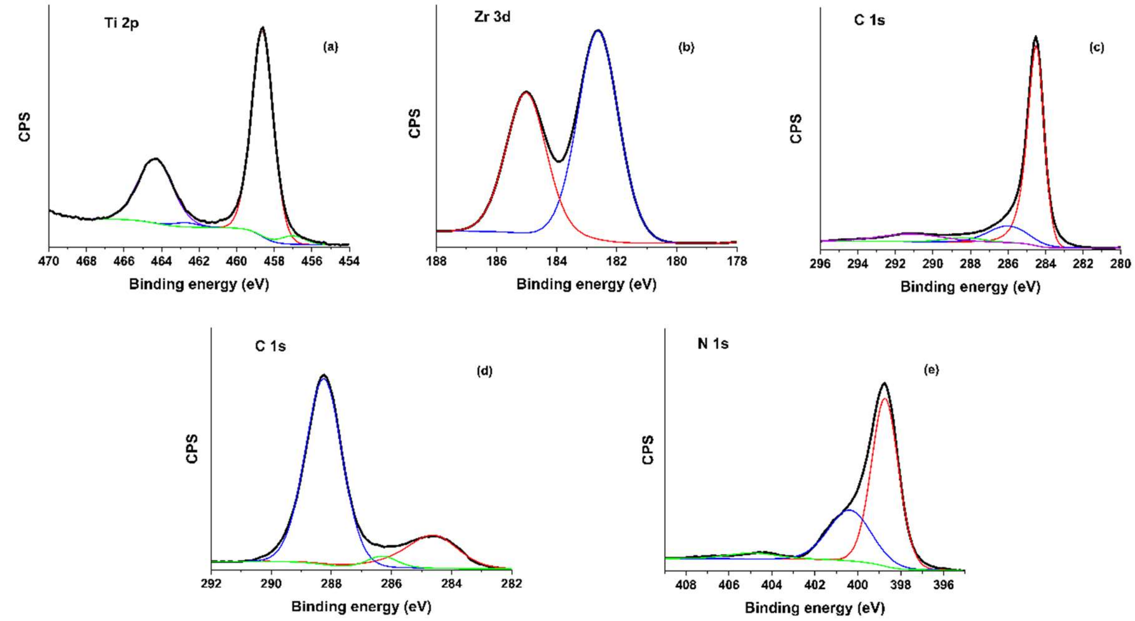

Figure S1. XPS regions for, ReO_x/TiO_2 (a) Ti 2p, ReO_x /ZrO_2: (a) Zr 3d, ReO_x /G200 (c) C 1s and ReO_x /g-C_3N_4 (d) C 1s and (e) N 1s

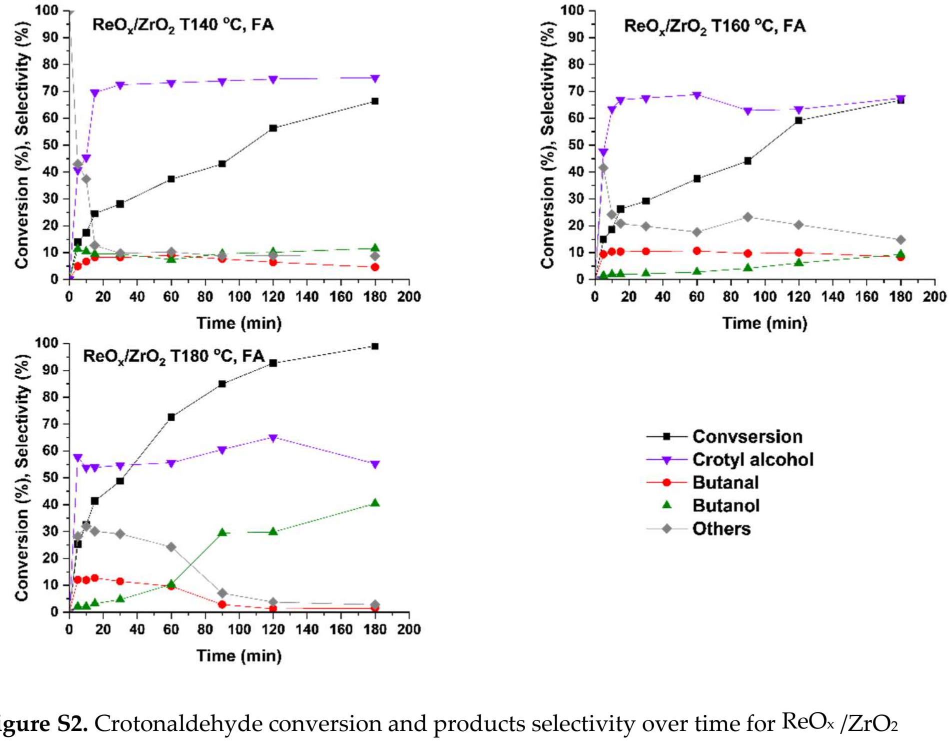

Figure S2. Crotonaldehyde conversion and products selectivity over time for ReO_x /ZrO_2

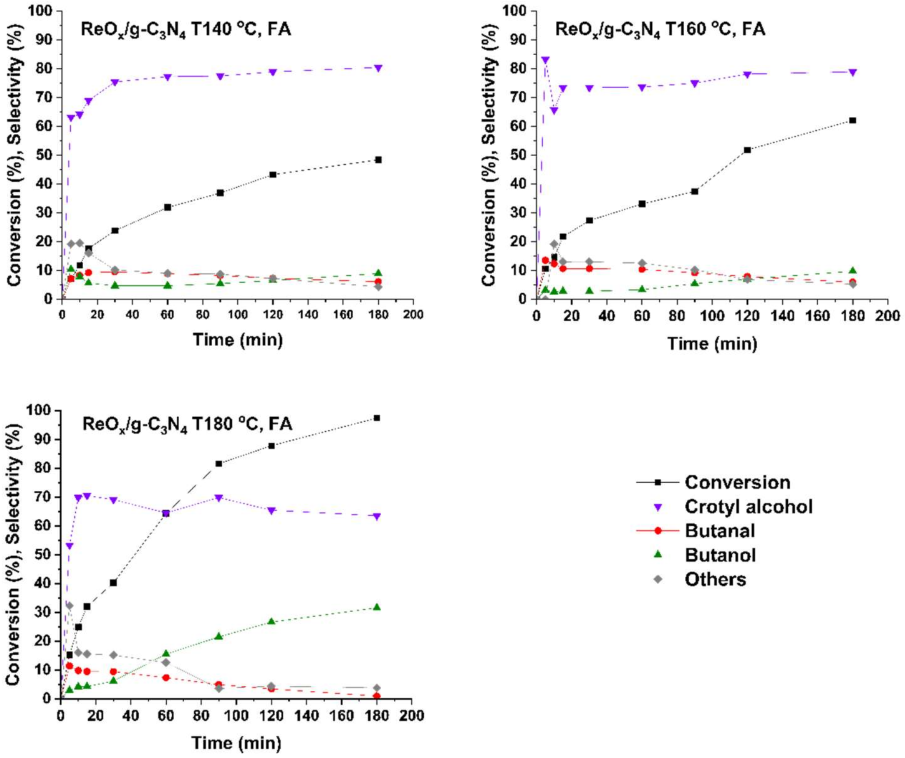

Figure S3. Crotonaldehyde conversion and products selectivity over time for ReO_x/g-C_3N_4

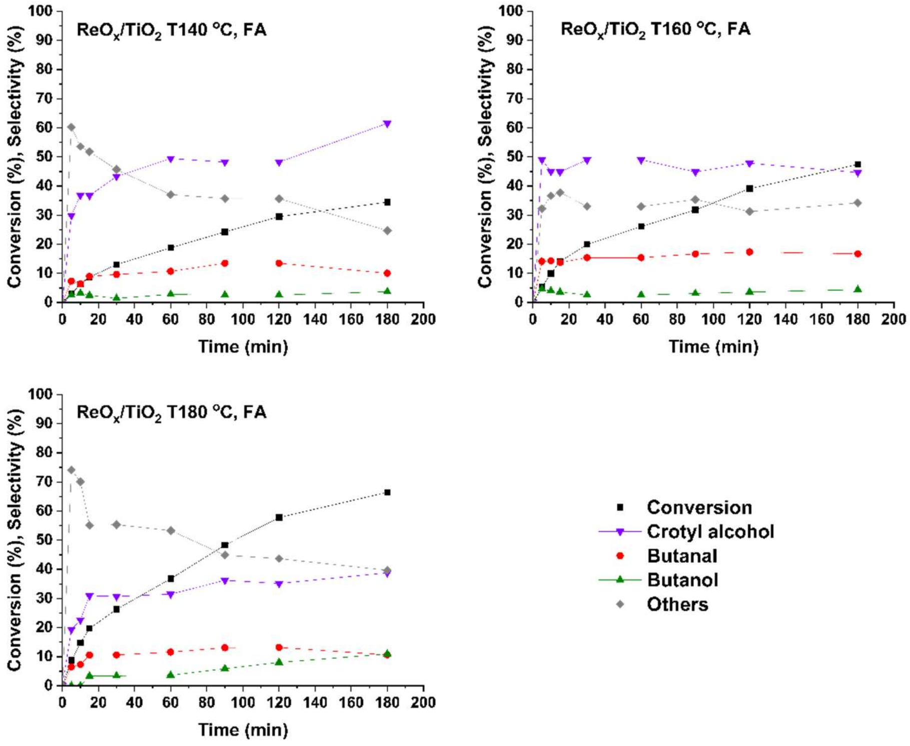

Figure S4. Crotonaldehyde conversion and products selectivity evolution with time for ReO_x/TiO_2

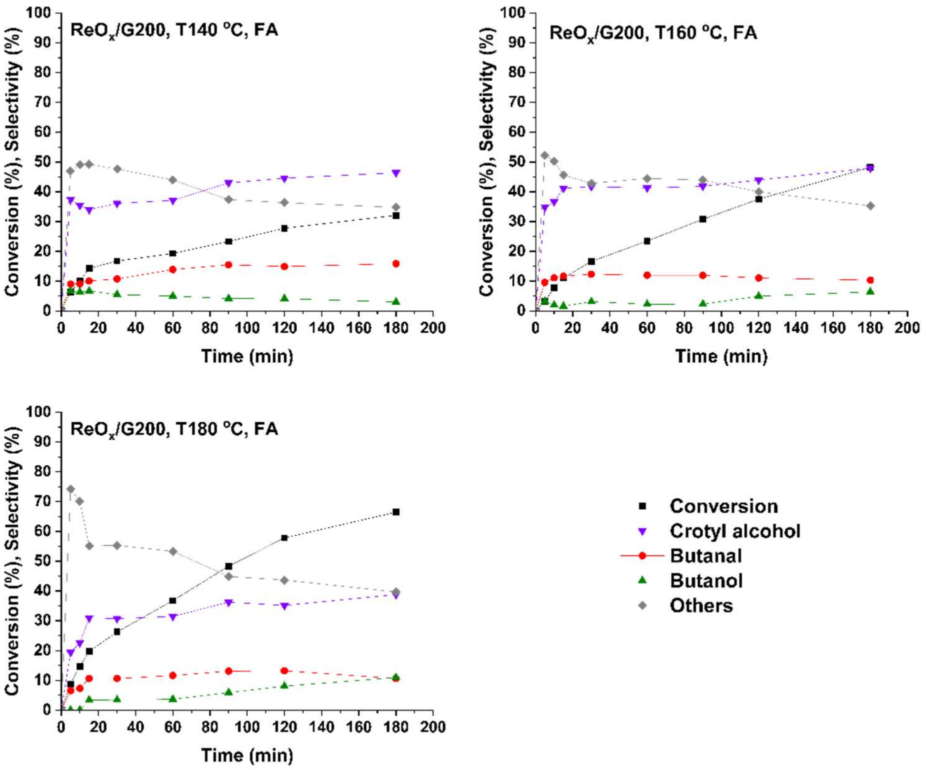

Figure S5. Crotonaldehyde conversion and products selectivity over time for ReO_x/G200

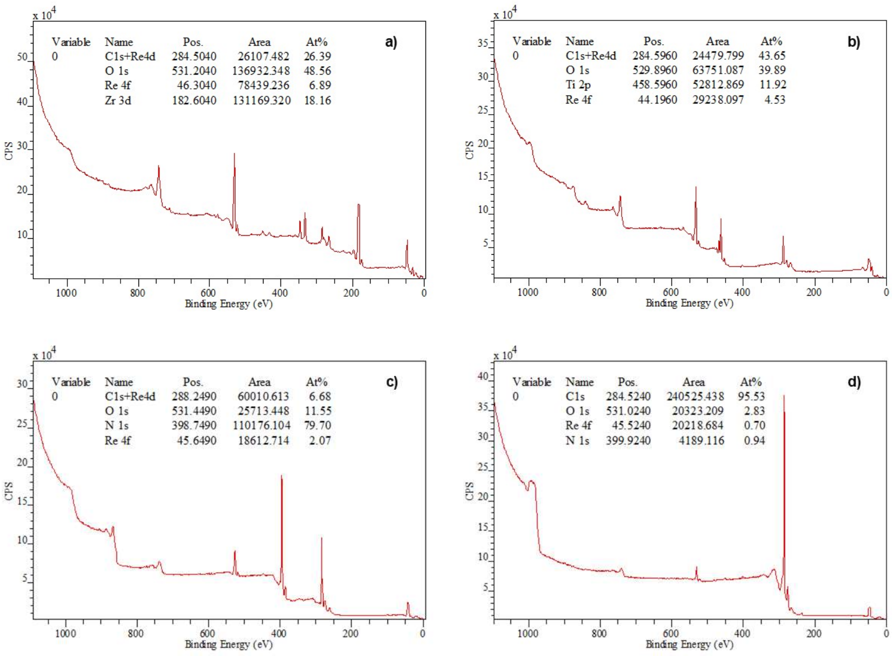

Figure S6. XPS survey spectrums for: a) ReO_x/ZrO_2, b) ReO_x/TiO_2, c) ReO_x/g-C_3N_4 and d) ReO_x/G200

Table S1. XPS parameters for all ReO_x supported catalysts

Catalyst	Element	Position	RSF[a]	FWHM[b]
ReO_x/ZrO_2	C 1s adv	284.56	1.00	1.42
	C 1s	286.22	1.00	2.5
	C 1s	289.02	1.00	1.68
	O 1s	531.65	2.5	2.85
	O 1s	530.61	2.85	1.95
	Re 4f $_{7/2}$ (VII)	46.33	6.46	1.34
	Re 4f $_{5/2}$ (VII)	48.74	5.08	1.37
	Re 4f $_{7/2}$ (VI)	45.67	6.46	2.5
	Re 4f $_{5/2}$ (VI)	48.08	5.08	2.28
	Re 4f $_{7/2}$ (IV)	43.10	6.46	2.28
	Re 4f $_{5/2}$ (IV)	45.51	5.08	2.00
	Zr 3d$_{5/2}$	182.61	4.33	1.58
	Zr 3d$_{3/2}$	185.01	2.97	1.57
ReO_x/TiO_2	C 1s adv	284.62	1.00	1.76
	C 1s	286.73	1.00	0.88
	C 1s	288.66	1.00	1.58
	O 1s TiO_2	529.89	2.85	1.56
	O 1s	531.81	2.85	2.50
	Re 4f $_{7/2}$ (VI)	45.70	6.46	1.55
	Re 4f $_{5/2}$ (VI)	48.11	5.08	1.62
	Re 4f $_{7/2}$ (IV)	43.5	6.46	2.39
	Re 4f $_{5/2}$ (IV)	45.9	5.08	2.50
	Re 4f $_{7/2}$ (II)	41.74	6.46	1.41
	Re 4f $_{5/2}$ (II)	44.15	5.08	1.53
	Ti 2p$_{3/2}$ (IV)	458.63	5.22	1.37
	Ti 2p$_{1/2}$ (IV)	464.33	2.68	2.17
	Ti 2p$_{3/2}$ (III)	457.00	5.22	1.50
	Ti 2p$_{1/2}$ (III)	462.70	2.68	1.50
$ReO_x/g\text{-}C_3N_4$	C 1s C-C	284.62	1.00	1.93
	C 1s	286.31	1.00	1.15
	C 1s N-C=N	288.25	1.00	1.44
	O 1s	531.00	2.85	1.46
	O 1s	533.14	2.85	2.80
	Re 4f $_{7/2}$ (VII)	46.61	6.46	2.08
	Re 4f $_{5/2}$ (VII)	49.02	5.08	2.02
	Re 4f $_{7/2}$ (VI)	45.59	6.46	1.42
	Re 4f $_{5/2}$ (VI)	48.00	5.08	1.37
	Re 4f $_{7/2}$ (IV)	43.48	6.46	2.32
	Re 4f $_{5/2}$ (IV)	45.89	5.08	2.00
	N 1s C-N=O	398.73	1.77	1.47
	N 1s C-N-H	400.37	1.77	2.32
	N 1s NO_2	404.90	1.77	2.71
ReO_x/G200	C1s graphite	284.50	1.00	0.97

	C 1s	286.00	1.00	2.50
	C 1s	288.33	1.00	2.50
	C 1s shake	291.16	1.00	3.98
	O 1s	530.84	2.85	1.78
	O 1s	532.38	2.85	2.77
	Re 4f 7/2 (VII)	46.73	6.46	2.24
	Re 4f 5/2 (VII)	49.14	5.08	2.50
	Re 4f 7/2 (VI)	45.52	6.46	1.64
	Re 4f 5/2 (VI)	47.93	5.08	1.62
	Re 4f 7/2 (IV)	42.90	6.46	1.53
	Re 4f 5/2 (IV)	45.31	5.08	1.00

[a]RSF: relative sensitivity factor

[b]FWHM: full width at half maximum
